# Oxidized low-density lipoprotein (oxLDL) supports *Mycobacterium tuberculosis* survival in macrophages by inducing lysosomal dysfunction

**DOI:** 10.1371/journal.ppat.1007724

**Published:** 2019-04-18

**Authors:** Frank Vrieling, Louis Wilson, Patrick C. N. Rensen, Gerhard Walzl, Tom H. M. Ottenhoff, Simone A. Joosten

**Affiliations:** 1 Department of Infectious Diseases, Leiden University Medical Center, Albinusdreef 2, ZA Leiden, The Netherlands; 2 Department of Medicine, Division of Endocrinology, Leiden University Medical Center, Albinusdreef 2, ZA Leiden, The Netherlands; 3 DST/NRF Center of Excellence for Biomedical Tuberculosis Research, SA MRC Center for TB Research, Division of Molecular Biology and Human Genetics, Department of Biomedical Sciences, Faculty of Medicine and Health Sciences Stellenbosch University, Francie van Zijl Drive, Tygerberg, Cape Town, South Africa; University of Massachusetts Medical School, UNITED STATES

## Abstract

Type 2 diabetes mellitus (DM) is a major risk factor for developing tuberculosis (TB). TB-DM comorbidity is expected to pose a serious future health problem due to the alarming rise in global DM incidence. At present, the causal underlying mechanisms linking DM and TB remain unclear. DM is associated with elevated levels of oxidized low-density lipoprotein (oxLDL), a pathologically modified lipoprotein which plays a key role during atherosclerosis development through the formation of lipid-loaded foamy macrophages, an event which also occurs during progression of the TB granuloma. We therefore hypothesized that oxLDL could be a common factor connecting DM to TB. To study this, we measured oxLDL levels in plasma samples of healthy controls, TB, DM and TB-DM patients, and subsequently investigated the effect of oxLDL treatment on human macrophage infection with *Mycobacterium tuberculosis* (*Mtb*). Plasma oxLDL levels were significantly elevated in DM patients and associated with high triglyceride levels in TB-DM. Strikingly, incubation with oxLDL strongly increased macrophage *Mtb* load compared to native or acetylated LDL (acLDL). Mechanistically, oxLDL -but not acLDL- treatment induced macrophage lysosomal cholesterol accumulation and increased protein levels of lysosomal and autophagy markers, while reducing *Mtb* colocalization with lysosomes. Importantly, combined treatment of acLDL and intracellular cholesterol transport inhibitor (U18666A) mimicked the oxLDL-induced lysosomal phenotype and impaired macrophage *Mtb* control, illustrating that the localization of lipid accumulation is critical. Collectively, these results demonstrate that oxLDL could be an important DM-associated TB-risk factor by causing lysosomal dysfunction and impaired control of *Mtb* infection in human macrophages.

## Author summary

Tuberculosis (TB) is an infectious disease of the lungs caused by a bacterium, *Mycobacterium tuberculosis* (*Mtb*), and is responsible for over a million deaths per year worldwide. Population studies have demonstrated that type 2 diabetes mellitus (DM) is a risk factor for TB as it triples the risk of developing the disease. DM is a metabolic disorder which is generally associated with obesity, and is characterized by resistance to the pancreatic hormone insulin and high blood glucose and lipid levels. As the global incidence of DM is rising at an alarming rate, especially in regions where TB is common, it is important to understand precisely how DM increases the risk of developing TB. Both TB and DM are associated with the development of foamy macrophages, lipid-loaded white blood cells, which can be the result of a specific lipoprotein particle called oxidized low-density lipoprotein (oxLDL). Here, we demonstrated that DM patients have high blood levels of oxLDL, and generating foamy macrophages with oxLDL supported *Mtb* survival after infection as a result of faulty intracellular cholesterol accumulation. Our results propose a proof of concept for oxLDL as a risk factor for TB development, encouraging future studies on lipid-lowering therapies for TB-DM.

## Introduction

Type 2 diabetes mellitus (DM) has been recognized as a major risk factor for tuberculosis (TB) for decades [1]. Recent epidemiological studies have demonstrated that DM triples the risk of developing active TB [2], and approximately 15% of global TB cases can be attributed to DM comorbidity [3]. The precise mechanisms through which DM enhances the risk of active TB disease progression are unknown, however it has been hypothesized that metabolic changes associated with DM attenuate the immune response towards *Mycobacterium tuberculosis* (*Mtb*), the causative pathogen of TB. As the global incidence of DM has been rising at an alarming rate [4], including more recently in TB endemic regions of African and Asia, it is of great importance to identify the molecular and cellular mechanisms underlying TB-DM comorbidity.

DM patients often suffer from dyslipidemia and oxidative stress, conditions which can contribute to the formation of oxidized low density lipoprotein (oxLDL) [5]. LDL can be oxidized by free radicals and reactive products of oxygenases, a process which has been mostly studied in the context of atherosclerosis during which oxLDL is generated in the subendothelial space of the arterial wall [6, 7]. High levels of circulating oxLDL were shown to be associated with DM, insulin resistance and decreased glucose tolerance [8–11]. oxLDL is recognized as a damage-associated molecular pattern (DAMP) by macrophages and is a ligand for various scavenger receptors on the cell surface, including CD36, scavenger receptor A (SR-A) and lectin-type oxidized LDL receptor 1 (LOX-1) [12]. The uptake of oxLDL by macrophages plays a major role during the pathophysiology of atherosclerosis as it leads to the generation of pro-inflammatory lipid-loaded foam cells in the arterial vessel wall [13, 14]. These macrophages exhibit increased scavenger receptor expression, cytokine secretion and production of oxidizing agents, supporting both immune cell infiltration and further generation of oxLDL which can culminate in atherosclerotic plaque formation [15].

Foamy macrophages also occur during TB progression and are thought to be of great importance for the development of TB granulomas and persisting *Mtb* infection, since the bacterium relies on host-derived lipids and cholesterol as a source of carbon for its survival [16–18]. Infection of alveolar macrophages with *Mtb* initiates the formation of the early TB granuloma, which progresses from a core of infected foam cells to an enclosed structure with a thick fibrous capsule and a lipid-rich caseous center of necrotic macrophages [16]. Various studies have demonstrated that *Mtb* and other mycobacteria are able to utilize host-derived lipids and even reprogram lipid metabolism in infected macrophages to induce foam cell formation, in part through the effects of mycobacterial cell wall lipids [19–24]. Interestingly, oxLDL was also found to accumulate in granulomas and alveolar macrophages of *Mtb* infected guinea pigs and to enhance bacterial replication [25], suggesting that local oxLDL production could play a role in foam cell formation and *Mtb* persistence during TB disease.

OxLDL-derived lipids have been demonstrated to be resistant to lysosomal esterases which are normally responsible for lipid breakdown. This results in lipid accumulation inside lysosomes after initial uptake by macrophages [26, 27], as well as to dysfunctions in the trafficking and efflux of intracellular cholesterol which mimic those observed in the lysosomal storage disorder Niemann Pick disease type C (NPC). During NPC disease, mutations in the lysosomal cholesterol transporters *NPC1* or *NPC2* result in severe neurological defects due to excessive intralysosomal storage of cholesterol and sphingolipids [28]. Cholesterol accumulation due to oxLDL uptake or NPC1-deficiency induces lysosomal dysfunction in macrophages, as it can interfere with phagolysosomal trafficking, maturation and fusion [29, 30]; inhibit autophagy [31, 32], an important cellular pathway which is simultaneously involved in lipid and cholesterol metabolism [33] and *Mtb* killing [34] in macrophages; increase lysosomal pH [35]; directly damage lysosomal membranes [36, 37]; and trigger various downstream inflammatory pathways such as formation of the NLRP3 inflammasome [38]. A recent paper demonstrated that both infection with live *M*. *smegmatis* or *M*. *bovis* BCG and treatment with mycobacterial cell wall lipids induced a NPC-like phenotype in macrophages with associated defects in lysosomal function [39], indicating that cholesterol accumulation could provide a permissive environment for mycobacteria in addition to being a nutritional source.

To investigate whether oxLDL is a molecular component in the interplay between TB and DM, we measured oxLDL concentrations in plasma samples of DM, TB and TB-DM patients and analyzed the effect of oxLDL on *in vitro Mtb* infection in primary human macrophages. We found that oxLDL is elevated in the plasma of DM patients and supported *Mtb* intracellular survival *in vitro* by inducing lysosomal dysfunction. Collectively, our findings provide a proof of concept for a contribution of oxLDL as a risk factor for TB during DM.

## Results

### Plasma oxLDL levels are increased in DM and TB-DM patients with dyslipidemia

First, we sought to confirm the presence of high levels of circulating oxLDL in DM patients from a TB endemic setting and to assess the relative impact of TB-DM comorbidity on circulating oxLDL levels. OxLDL concentrations were determined in plasma samples from healthy endemic controls (HC), TB, DM and TB-DM patients of a South-African cohort, previously used in a lipidomic biomarker analysis [40], by sandwich ELISA using a monoclonal antibody against a conformational epitope in oxidized ApoB-100 [41]. Patient characteristics are described in S1 Table.

**Fig 1:**
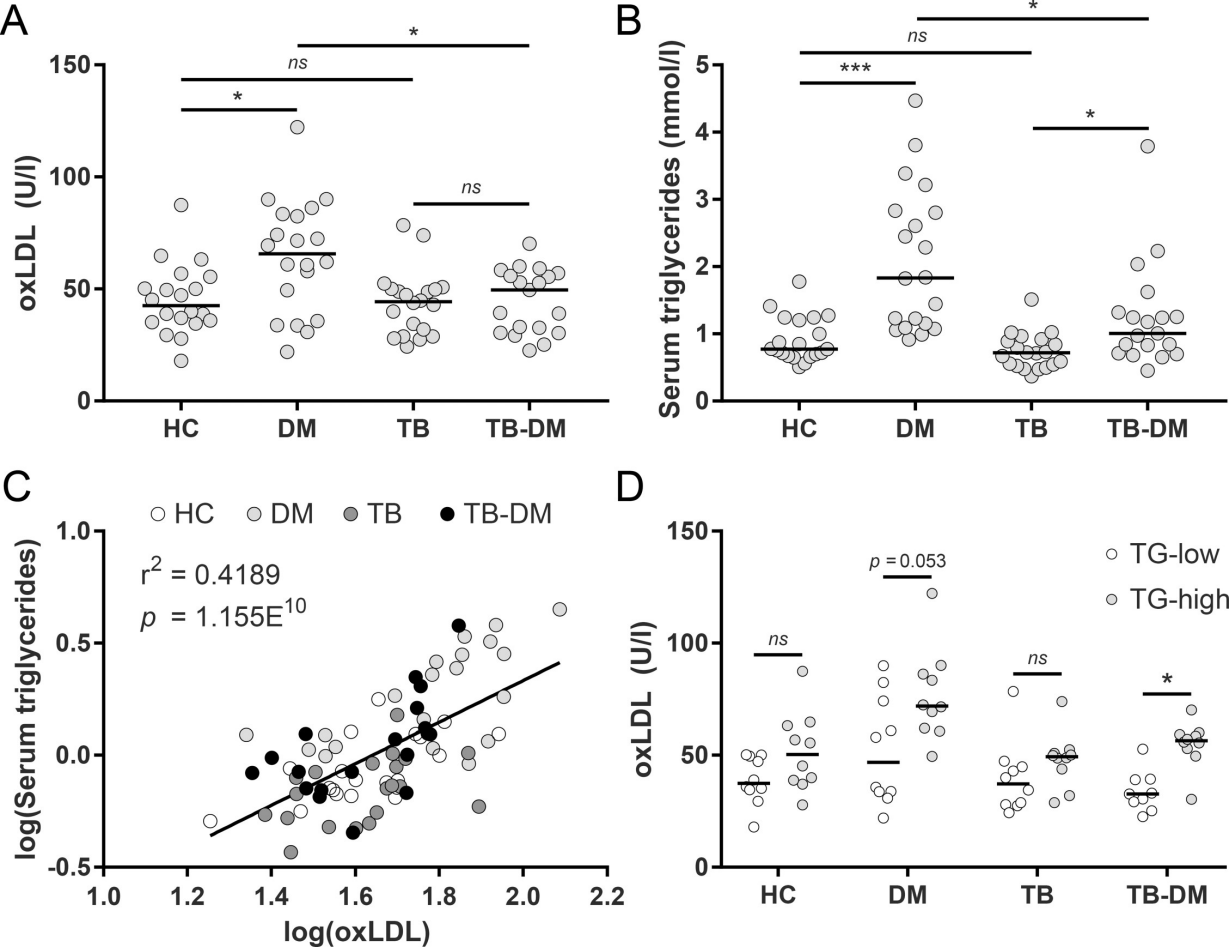
OxLDL levels are increased in DM and associated with triglyceride levels in TB-DM patients. (A) OxLDL concentrations (U/l) were determined in plasma samples of healthy controls (HC) (n = 20), TB (n = 20), DM (n = 20) and TB-DM patients (n = 19) by ELISA. (B) Serum triglyceride (TG) levels (mmol/l) were determined by H^+^-NMR spectroscopy. (C) Linear correlation analysis of log-transformed serum triglyceride and oxLDL levels. (D) Plasma oxLDL concentrations in HC, TB, DM and TB-DM patients stratified by TG levels (TG-high vs TG-low; n = 10/group except for TB-DM + TG-low: n = 9). Individual patients are depicted as dots with group medians. Statistical significance was determined by Kruskal-Wallis test with post-hoc Dunn’s test. * = *p* < 0.05, *** = *p* < 0.001.

Plasma oxLDL levels were significantly higher in DM patients (median: 65.8 [interquartile range: 39.2–83.2] U/l) compared to both HC (42.3 [35.3–82.2] U/l, *p* < 0.05) and TB-DM patients (44.4 [30.3–56.7] U/l, *p* < 0.05) (Fig 1A), but not significantly different in patients with TB-DM compared to TB alone (44.3 [29.6–50.0] U/l). However, a clear dichotomy was distinguishable in the TB-DM patient group: our previous analysis of these samples [40] had demonstrated that both DM and TB-DM patients displayed characteristics of dyslipidemia, as evidenced by high levels of serum triglycerides (TG) (Fig 1B). Furthermore, serum triglyceride levels were positively correlated with oxLDL across all measured samples (r^2^: 0.4189, *p* = 1.155^−10^) (Fig 1C). To investigate whether oxLDL levels were related to the severity of dyslipidemia in TB-DM patients, we subdivided the groups according to serum TG-concentrations (TG-high and TG-low, Fig 1D). DM and TB-DM patients with TG-high had increased oxLDL levels compared to those with TG-low (DM: 72.0 [61.7–87.1] vs 46.8 [33.0–76.3] U/l, *p* = 0.053; TB-DM: 56.4 [52.1–59.4] vs 32.7 [27.2–39.2] U/l, *p* < 0.05). Taken together, the results validate that DM patients have increased levels of circulating oxLDL and that plasma oxLDL concentrations are elevated in DM and TB-DM patients with concomitant hypertriglyceridemia.

### OxLDL treatment increases *Mtb* bacterial burden in infected human macrophages

**Fig 2:**
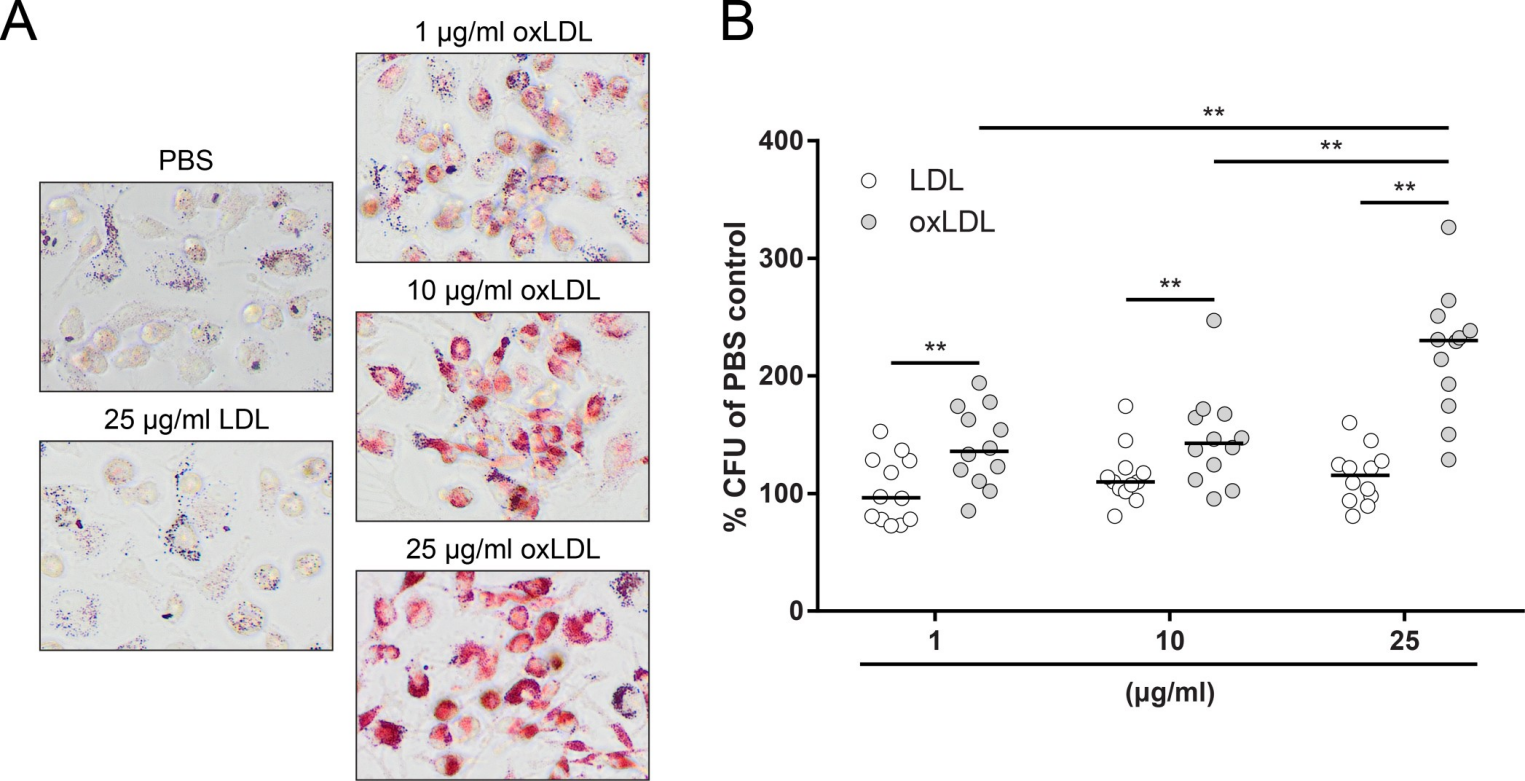
OxLDL-treated macrophages have an increased *Mtb* burden. Primary human macrophages were treated with PBS control, native LDL or oxLDL (1, 10 or 25 μg/ml) overnight and subsequently infected with *Mtb* H37Rv at a MOI of 10:1. (A) Oil Red O staining of macrophages treated overnight with PBS, 25 μg/ml LDL or 1, 10 and 25 μg/ml oxLDL. Pictures were taken at a 20x magnification. (B) Macrophages were lysed at 24 h post-infection and bacterial load was determined by CFU assay. Results were normalized versus PBS control (n = 8). Individual donors are depicted as dots with group medians. Statistical significance was determined by Wilcoxon signed rank test with post-hoc FDR correction. ** = *p* < 0.01.

As oxLDL was clearly elevated in DM patients and has been described to have profound effects on macrophage function, we hypothesized that oxLDL treatment could compromise the capacity of macrophages to control *Mtb* infection. To investigate this, macrophages were treated with 1, 10 or 25 μg/ml oxLDL or native LDL overnight. Oil Red O staining indicated a dose-dependent increase in intracellular lipid levels after oxLDL treatment, while native LDL did not induce foam cells (Figs 2A and S1B). These macrophages were subsequently infected for 24 h with *Mtb* H37Rv and intracellular bacterial loads were assessed by bacterial colony forming unit (CFU) assay. OxLDL treatment significantly increased *Mtb* load compared to native LDL at all tested concentrations (1 μg/ml: 136% [113% - 171%] vs 97% [78% - 128%], *p* < 0.01; 10 μg/ml: 143% [115% - 167%] vs 110% [102% - 121%], *p* < 0.01; 25 μg/ml: 230% [179% - 248%] vs 115% [94.8% - 127%], *p* < 0.01), and this effect was dose-dependent (25 μg/ml oxLDL vs 1 μg/ml: *p* < 0.01; vs 10 μg/ml: *p* < 0.01) (Fig 2B). The magnitude of the increase in bacterial load was not correlated with small fluctuations in infectious load (MOI) (S1C Fig).

**Fig 3:**
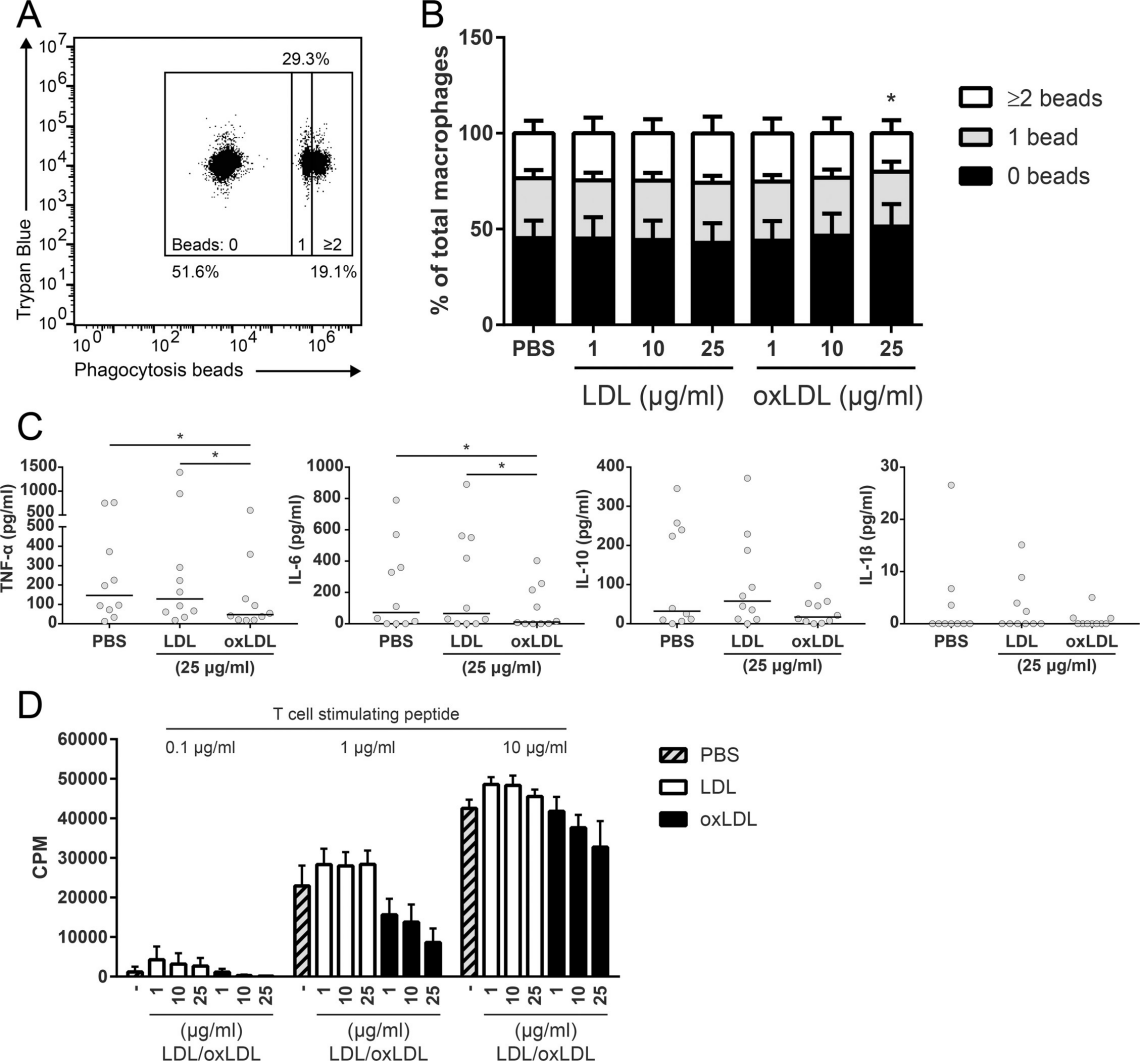
Functional analysis of oxLDL-treated macrophages. Primary human macrophages were treated with PBS control, native LDL or oxLDL (1, 10 or 25 μg/ml) overnight. (A) Macrophages were incubated with fluorescent phagocytosis beads at a MOI of 10:1 and subsequently analyzed by flow cytometry. Fluorescence of extracellular beads was quenched with Trypan Blue. (B) Percentage of macrophages with 0, 1 or ≥2 beads internalized beads (n = 6). Data is represented as means with standard deviations. (C) Macrophages were infected with *Mtb* H37Rv at a MOI of 10:1 for 1 h. Supernatants were harvested at 24 h post-infection and concentrations of TNF-α, IL-6, IL-10 and IL-1β were determined by ELISA (n = 10). Individual donors are depicted as dots with group medians. (D) Macrophages were co-cultured for four days with the HLA-DR2-restricted CD4^+^ T cell R2F10 at a ratio of 1:4 and 0.1, 1 or 10 μg/ml of its cognate peptide. T cell proliferation was measured by tritium-thymidine incorporation during the last 24 h (n = 3). Data is represented as means with standard deviations. Statistical significance was determined by Wilcoxon signed rank test with post-hoc FDR correction. * = *p* < 0.05.

While these experiments demonstrated that oxLDL treatment supported *Mtb* persistence in human macrophages, it was unclear whether this was the result of increased phagocytosis,reduced intracellular mycobacterial control or enhanced replication. To gain a better understanding on the cellular processes affected by oxLDL treatment, we explored the functional consequences of oxLDL-induced foam cell formation. Firstly, the phagocytic capacity of oxLDL-treated macrophages was assessed to investigate whether the increased mycobacterial load might be related to enhanced *Mtb* uptake. Macrophages treated with either native LDL or oxLDL were incubated with fluorescent polystyrene beads and bead phagocytosis was quantified by flow cytometry (Fig 3A). Although a small but significant decrease in bead uptake was observed in macrophages incubated with 25 μg/ml oxLDL compared to LDL (*p* < 0.05) (Fig 3B), overall macrophage phagocytic capacity was unaffected by oxLDL treatment, indicating that the increased mycobacterial burden in oxLDL-derived foam cells was probably not the result of increased phagocytic uptake. To confirm this, we investigated the intracellular bacterial load of oxLDL-treated macrophages directly after 1 h of infection and found no significant differences compared to control conditions (S2C Fig).

Next, we explored the cytokine response of oxLDL-derived foam cells to *Mtb*-infection as earlier studies had reported potent oxLDL-induced pro-inflammatory cytokine production. In contrast to these studies, oxLDL-treatment in our experiments significantly decreased the secretion of TNF-α compared to treatment with LDL (47 [20 – 186] vs 128 [54 – 453] pg/ml, *p* < 0.05) or PBS (146 [62 – 466] pg/ml, *p* < 0.05). Similar results were obtained for IL-6 after oxLDL treatment versus LDL (11 [0–226] vs 66 [0–553] pg/ml, *p* < 0.05) or PBS (73 [0–412] pg/ml, *p* < 0.05), although some inter-individual variation was observed (Fig 3C). IL-10 levels were not significantly affected by oxLDL, while IL-1β levels were very low.

Finally, oxLDL-derived macrophages were co-cultured with a HLA-DR2-restricted CD4^+^ T cell clone (R2F10) and its cognate peptide (*Mlep* hsp65 p418–427) and T cell proliferation was measured to determine macrophage dependent antigen presentation. OxLDL treatment dose-dependently diminished the antigen presentation capacity of macrophages, especially at suboptimal peptide concentrations (Fig 3D). Similar results were obtained using a second, HLA-DR3-restricted CD4^+^ T cell clone (Rp15 1–1) (S2A Fig), both after loading with its cognate peptide or purified protein derivative (PPD). This diminished antigen presentation capacity was independent of cell surface expression of HLA-DR and co-stimulatory molecules CD80 and CD86 (S2B Fig). Taken together, oxLDL treatment impaired several macrophage functions, including antigen presentation and pro-inflammatory cytokine secretion, but not their phagocytic capacity.

### OxLDL supports *Mtb* intracellular survival through lysosomal cholesterol accumulation

**Fig 4:**
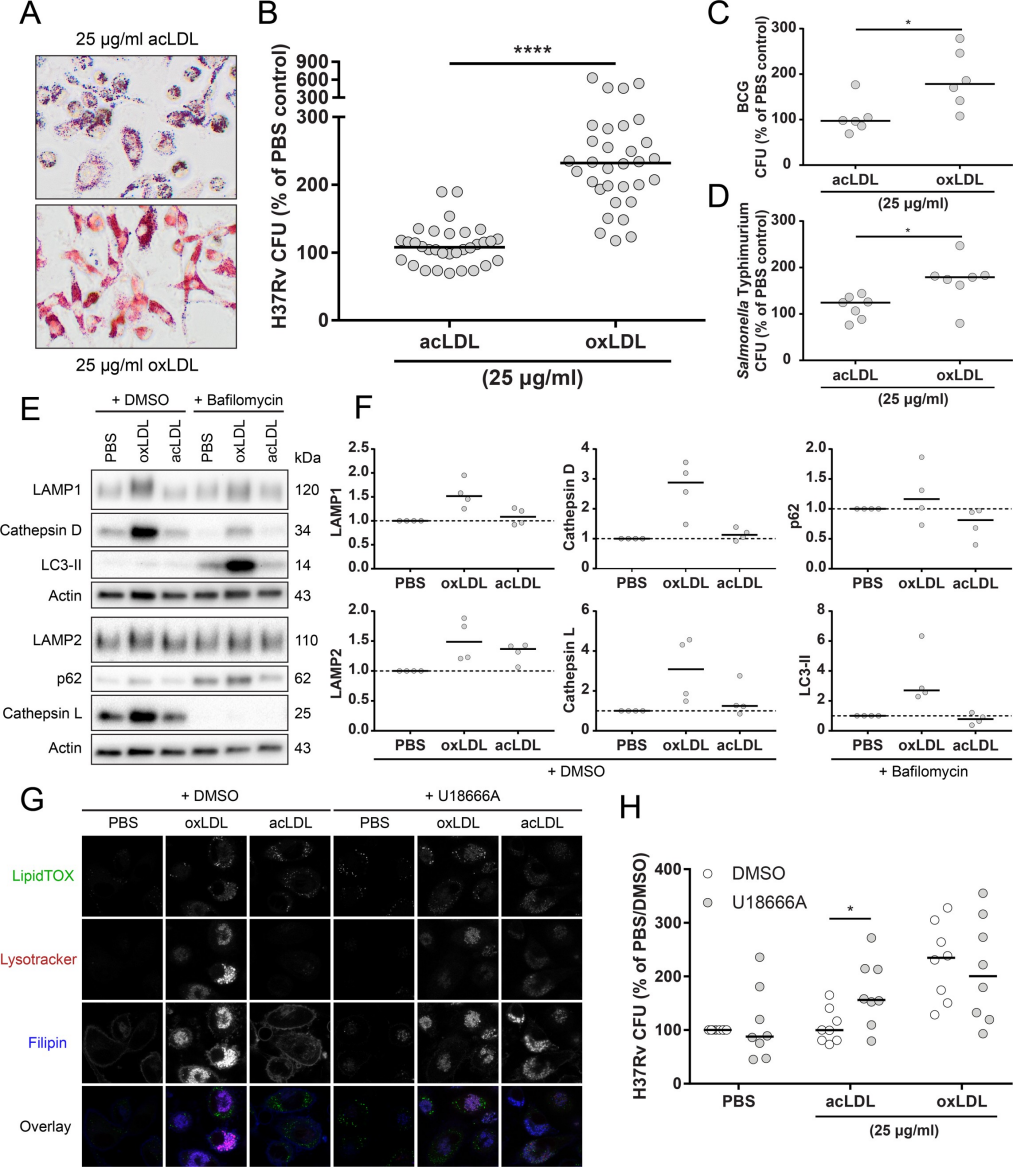
Lysosomal cholesterol accumulation attenuates macrophage *Mtb* control. Primary human macrophages were treated with PBS control, acLDL or oxLDL (25 μg/ml) overnight. (A) Oil Red O staining of macrophages treated overnight with 25 μg/ml acLDL or oxLDL. Pictures were taken at a 20x magnification. Macrophages were infected with *Mtb* H37Rv (B) (n = 32), *M*. *bovis* BCG (C) (n = 6) or *Salmonella enterica* serovar Typhimurium (D) (n = 6) at a MOI of 10:1. Cells were lysed at 24 h post-infection and bacterial load was determined by CFU assay. Results were normalized versus PBS control. (E) Western blot analysis of lysosomal and autophagy markers in macrophages treated with bafilomycin A1 (10 nM) or DMSO control during 24 h of H37Rv *Mtb* infection. Data shown is from one representative donor (n = 4). (F) Quantification of LAMP1, LAMP2, Cathepsin D, Cathepsin L (+ DMSO), p62 and LC3-II (+ bafilomycin A1) protein levels. Protein levels were first normalized to actin and subsequently versus PBS control (n = 4). (G) Macrophages were co-treated with U18666A (3 μg/ml) or DMSO control for 24 h. Cells were subsequently stained for neutral lipids (LipidTOX, green), lysosomes (Lysotracker, red) and cholesterol (filipin, blue) and analyzed by confocal microscopy. Pictures were taken at a 63x magnification. Scale bars represent 5 μM. (H) Macrophages were co-treated with U18666A (3 μg/ml) or DMSO control for 24 h pre- and post-infection with *Mtb* H37Rv at a MOI of 10:1. Cells were lysed at 24 h post-infection and bacterial load was determined by CFU assay. Results were normalized versus PBS control (+ DMSO). Individual donors are depicted as dots with group medians. Statistical significance was determined by Wilcoxon signed rank test. * = *p* < 0.05, **** = *p* < 0.0001.

OxLDL-derived free and esterified cholesterol have been demonstrated to be sequestered in lysosomes in macrophages [26, 27], which potentially leads to lysosomal dysfunction. To investigate whether lysosomal localization of oxLDL lipids is required for its effect on *Mtb* load, oxLDL treatment was compared to acLDL, a non-naturally occurring modified lipoprotein which is endocytosed through identical scavenger receptor pathways as oxLDL, but does not induce lysosomal cholesterol accumulation [26, 42]. In resemblance to oxLDL, acLDL treatment of macrophages resulted in foam cell formation. However, while lipid staining intensities were similar (S1B Fig), clear differences in intracellular lipid localization and droplet structure were observed between both types of lipoproteins: in general, acLDL-induced intracellular lipid droplets were darker in color and appeared more granular than those resulting from oxLDL treatment (Fig 4A). Most importantly, however, acLDL did not affect macrophage *Mtb* load compared to untreated macrophages while oxLDL treatment significantly increased mycobacterial load (Fig 4B: oxLDL: 232% [194%– 278%] vs acLDL: 108% [88% - 126%]; *p* < 0.0001). This effect was not restricted to *Mtb*, as comparable results were obtained after macrophage infection with *Salmonella enterica* serovar Typhimurium (*Stm*) (Fig 4D: 179% [162% - 183%] vs 124% [88% - 136%]; *p* < 0.05) and *M*. *bovis* BCG (Fig 4C: 178% [133% - 254%] vs 97% [82% - 123%]; *p* < 0.05). To examine whether the observed difference between oxLDL and acLDL could be related to lysosomal function, their effect on lysosomal and autophagy markers during *Mtb* infection was analyzed by Western blot (Fig 4E). OxLDL treatment increased protein levels of lysosomal markers compared to PBS and acLDL, as demonstrated by higher levels of lysosomal membrane glycoproteins (LAMP1 & LAMP2) and proteases (Cathepsin D & L) (Fig 4F), also including the 48 kDa processing intermediate pro-cathepsin D (S3A Fig). Furthermore, oxLDL but not acLDL treatment led to an increased accumulation of LC3-II in the presence of vacuolar type H^+^-ATPase inhibitor bafilomycin A1 (10 nM) to block vesicle breakdown, indicative of increased autophagic flux. In contrast, levels of autophagosome cargo protein p62, a mediator of selective autophagy, were not elevated by oxLDL (Fig 4F). Collectively, these results indicate that oxLDL induces a general defect in macrophage antimicrobial function which is dependent on intracellular lipid localization.

To further substantiate this hypothesis, macrophages were treated with PBS, oxLDL or acLDL in the absence or presence of U18666A (3 μg/ml), an inhibitor of intracellular cholesterol transport [43]. Lysosomal cholesterol sequestration was visualized using confocal microscopy by staining with fluorescent probes for neutral lipids (LipidTOX), lysosomes (Lysotracker) and cholesterol (filipin) (Fig 4G). OxLDL treatment induced a marked accumulation of cholesterol inside lysosomal vesicles as indicated by filipin and Lysotracker colocalization, which was not observed in macrophages treated with PBS or acLDL. Strikingly, when combined with U18666A, acLDL-treated macrophages showed identical lysosomal cholesterol sequestration as oxLDL. The absence of an effect of acLDL treatment alone on *Mtb* load suggested that the localization of cholesterol inside lysosomes might be a causative factor in the increased *Mtb* growth phenotype of oxLDL-treated macrophages. To test this, we investigated whether combined treatment of acLDL with U18666A could mimic the effect of oxLDL on macrophage *Mtb* control. Indeed, while U18666A alone or in combination with oxLDL did not significantly alter macrophage phenotype (Fig 4G) and *Mtb* load, it increased mycobacterial burden when applied in conjunction with acLDL compared to DMSO control (Fig 4H: 169% ± 62% vs 107% ± 32%; *p* < 0.05). Similar to oxLDL, U18666A treatment alone and in combination with acLDL increased protein levels of lysosome and autophagy markers in *Mtb*-infected macrophages, most notably Cathepsin L and when combined with bafilomycin A1 (10 nM), p62 and LC3-II (S3B Fig). Macrophage viability was unaffected by oxLDL and/or U18666A treatment in combination with *Mtb* infection as determined by combined Hoechst/propidium iodide (PI) staining (S3C and S3D Fig). Collectively, these results indicate that not simply the presence, but the specific accumulation of cholesterol inside lysosomes is crucial for the oxLDL- and U18666A-induced increase in *Mtb* survival in human macrophages.

While the above model proposes that oxLDL can interfere with macrophage mycobacterial control, we could not yet exclude whether oxLDL-induced foam cell formation also supported *Mtb* replication, possibly by providing increased nutrients. To gain a better understanding of overall kinetics of oxLDL-induced increased *Mtb* load and its associated cytokine response, infected macrophages treated with PBS control, oxLDL or acLDL were infected with *Mtb* and the intracellular bacterial load and concentrations of 29 cytokines and chemokines in supernatants were determined at 0 (uptake control), 4, 24, 48, 72 and 144 h post-infection. OxLDL treatment showed increased *Mtb* survival compared to PBS as early as 4 h post-infection, and versus both PBS and acLDL at all later time points (24–144 h) (S4A Fig). For all treatment conditions the intracellular *Mtb* load decreased with time, ranging from 1.3 to 12.4% of original bacterial uptake after 144 h of infection, which is supportive of a model in which the effect of oxLDL is the result of inhibited bacterial killing and not of increased bacterial outgrowth.

The multiplex results were congruent with the ELISA data from Fig 3C, as oxLDL-treated macrophages produced significantly lower levels of TNF-α and IL-6 after 24 h of *Mtb* infection compared to PBS control (S4B Fig). Many cyto- and chemokine concentrations were lower in oxLDL-treated macrophages between 4–48 h of *Mtb* infection, while supernatants from acLDL-treated macrophages often showed intermediate levels compared to PBS and oxLDL (IL-10, IL-6, TNF-α, IL-8, CCL3, CCL4, G-CSF, GM-CSF). We did not find significant differences at 72 and 144 h post-infection after FDR correction. IL-1RA was the only cytokine which showed increased production as a result of oxLDL, although the magnitude of this response varied between donors. Concentrations of CXCL10, IFNα2, CCL2 and VEGF increased as a result of *Mtb* infection, however no differences were observed between treatment conditions for these factors. Levels of Epidermal Growth Factor (EGF), Eotaxin, IFNγ, IL-12p40, IL-12p70, IL-1β, IL-13, IL-15, IL-17A, IL-1α, IL-2, IL-3, IL-4, IL-5, IL-7 and TNF-β were measured but not shown as their concentrations were either very low in all samples (<100 pg/ml) or not detectable. Taken together, these experiments provide further evidence for an overall diminished cytokine response as a result of oxLDL treatment during *Mtb* infection.

### OxLDL-inhibited mycobacterial killing is not rescued by small-molecules targeting known downstream signaling pathways

**Fig 5:**
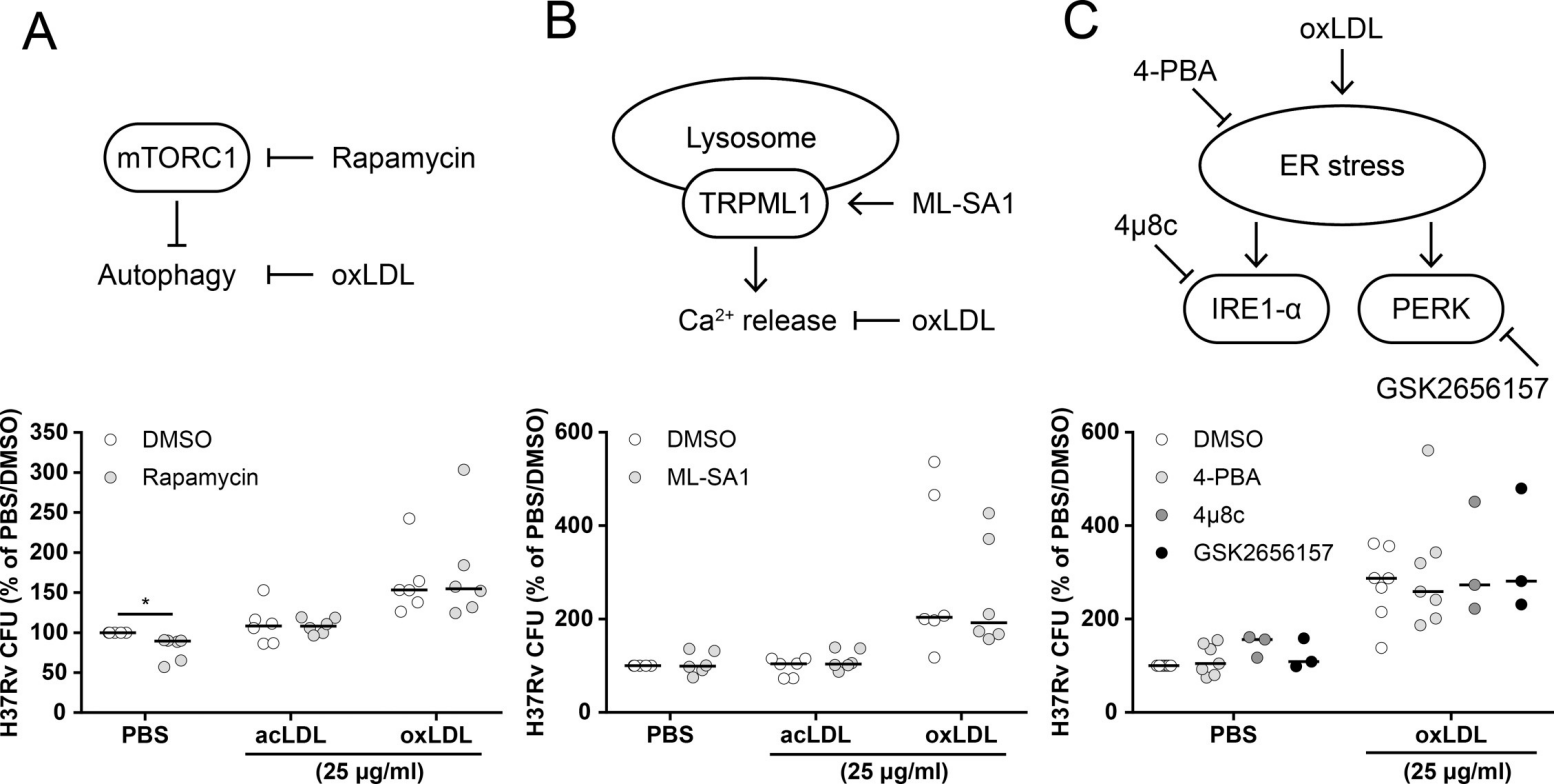
oxLDL-induced *Mtb* survival was not reversed by targeting known downstream pathways. Primary human macrophages were treated with PBS control, acLDL or oxLDL (25 μg/ml) overnight and subsequently infected with *Mtb* H37Rv at a MOI of 10:1 and treated with compounds or DMSO control overnight. Cells were lysed at 24 h post-infection and bacterial load was determined by CFU assay. The following treatments were applied: (A) rapamycin (2 μM, n = 6) to induce mTORC1-regulated autophagy, (B) ML-SA1 (10 μM, n = 6) to stimulate lysosomal Ca^2+^ release and (C) 4-PBA (3 mM, n = 7), 4μ8c (10 μM, n = 3) and GSK2656157 (10 μM, n = 3) to inhibit the ER stress response. Individual donors are depicted as dots with group medians. Results were normalized versus PBS control (+ DMSO). Statistical significance was determined by Wilcoxon signed rank test. * = *p* < 0.05.

To identify the relevant molecular processes which are deregulated by lysosomal cholesterol accumulation, oxLDL-treated macrophages infected with *Mtb* were treated with compounds targeting various cell signaling pathways which are known to be affected by oxLDL in an attempt to rescue their antimicrobial capacity. Firstly, infected foamy macrophages were treated with rapamycin, an inhibitor of mammalian target of rapamycin complex 1 (mTORC1). mTOR is a master regulator of various cellular pathways including autophagy, and rapamycin-induced autophagy was reported to ameliorate foam cell formation [44, 45]. Rapamycin (2 μM) slightly but significantly reduced *Mtb* load compared to DMSO in PBS-treated macrophages (80 ± 15% of PBS/DMSO, *p* < 0.05), but did not affect bacterial burden in either oxLDL or acLDL-induced foamy macrophages (Fig 5A). Secondly, lysosomal storage disorders such as NPC disease are associated with defects in lysosomal Ca^2+^ homeostasis [46], and activation of the lysosomal ion channel transient receptor potential channel 1 (TRPML1) by small-molecule activator ML-SA1 was shown to rescue lysosomal trafficking in NPC^-/-^-macrophages [30]. However, ML-SA1 treatment (10 μM) did not affect *Mtb* infection in any of our conditions (Fig 5B). Finally, oxLDL can induce endoplasmic reticulum (ER) stress in macrophages [47], a state of disturbed ER homeostasis due to accumulation of unfolded proteins and/or disrupted Ca^2+^ handling which plays a role in the apoptotic response in atherosclerotic plaques and the TB granuloma [48, 49]. Treatment of *Mtb*-infected macrophages with three established reducers of the ER stress response, namely chemical chaperone 4-phenylbutyrate (4-PBA; 3 mM) and downstream kinase inhibitors 4μ8c (10 μM) and GSK2656157 (10 μM) (respectively targeting inositol-requiring enzyme 1-α (IRE1-α) and protein kinase RNA-like endoplasmic reticulum kinase (PERK)), did not alleviate the oxLDL-induced increase in mycobacterial survival (Fig 5C). In conclusion, chemical modulation of mTOR signaling, lysosomal Ca^2+^ homeostasis or ER stress did not reverse the oxLDL-induced increased mycobacterial load in human macrophages.

### OxLDL inhibits *Mtb* localization to functional lysosomes in infected macrophages

**Fig 6:**
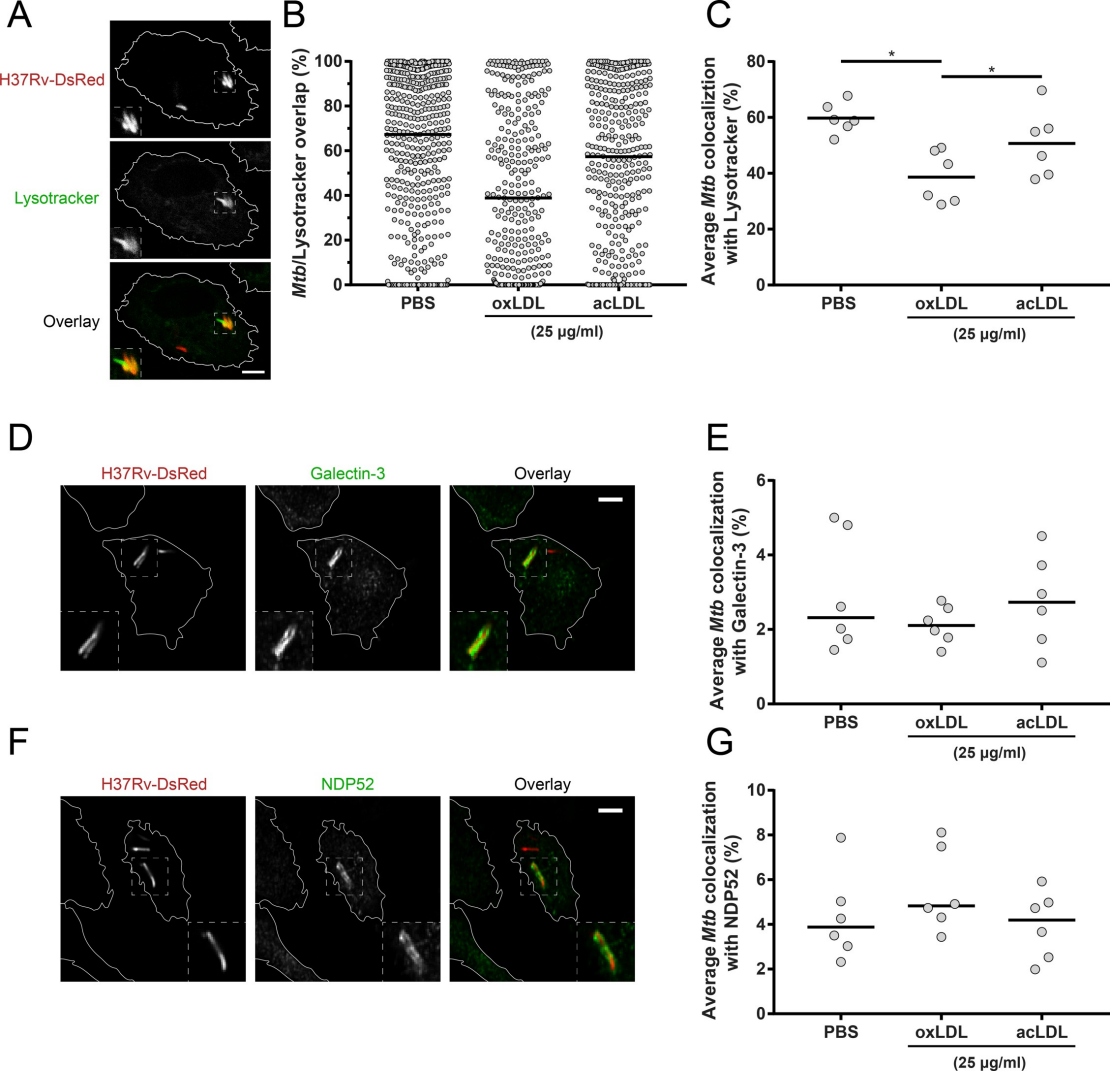
oxLDL impairs *Mtb* localization to lysosomes in macrophages. Primary human macrophages were treated overnight with PBS control, acLDL or oxLDL (25 μg/ml) and subsequently infected with DsRed-*Mtb* H37Rv (red) at a MOI of 10:1. Cells were stained (green) for lysosomes (Lysotracker) (A), galectin-3 (D) or NDP52 (F) at 4 h post-infection and analyzed by confocal microscopy. Pictures were taken at a 63x magnification. Scale bars represent 5 μM. Percentage overlap of intracellular mycobacteria with staining was determined for 3 wells * 3 = 9 pictures per condition. (B) Results of a representative donor of *Mtb* overlap with Lysotracker. Individual mycobacteria are represented by dots with group medians. Average colocalization of *Mtb* with Lysotracker (C), galectin-3 (E) and NDP52 (G) are displayed for macrophages from six independent donors. Individual donors are depicted as dots with group medians. Statistical significance was determined by Wilcoxon signed rank test with post-hoc FDR correction. * = *p* < 0.05.

The above experiments demonstrated that the endolysosomal system is pivotal for oxLDL-induced increased mycobacterial survival. As earlier studies have reported that cholesterol accumulation impaired proper lysosomal trafficking [29, 30], we hypothesized that *Mtb* trafficking to functional lysosomes was inhibited by oxLDL treatment. To investigate this, oxLDL-treated macrophages infected with fluorescent DsRed-expressing H37Rv were stained for functional lysosomes with Lysotracker (Fig 6A), and lysosomal colocalization was determined for each intracellular mycobacterium individually (Fig 6B). OxLDL significantly decreased the average colocalization between *Mtb* and Lysotracker (39 ± 9%) compared to acLDL (51 ± 12%, *p* < 0.05) or PBS treatment (60 ± 6%, *p* < 0.05) (Fig 6C), indicating that oxLDL inhibits phagolysosomal fusion in *Mtb*-infected macrophages.

In an attempt to identify the specific lysosomal pathways affected by oxLDL treatment, we investigated colocalization of *Mtb* with galectin-3 and NDP52. Galectins are carbohydrate-binding proteins which play a role in targeting damaged endomembrane structures for autophagy [50], including phagolysosomes damaged by *Stm* or *Mtb* [51–53], and galectin-3 colocalization with lysosomes is an established measure of lysosomal damage [54]. NDP52 is an autophagy adaptor which has previously been implicated in the autophagic clearance of both *Stm* and *Mtb* [51, 55, 56]. Although colocalization events with *Mtb* were observed for both galectin-3 and NDP52, this occurred for a minority of intracellular bacteria (range 2–8% of bacteria) and no significant differences were found between oxLDL and control conditions (Fig 6D–6G).

## Discussion

The looming epidemic of concurrent TB-DM poses a serious global health problem. Identification of the causal molecular and cellular mechanisms underlying the increased risk of TB in DM patients is paramount for adequate treatment. Previously, we have demonstrated that TB-DM patients have a blood lipid profile with pro-atherogenic properties [40], which could have implications for TB-DM pathogenesis. We now identify oxLDL as a potential risk factor for TB. OxLDL levels were found to be increased in plasma samples of DM patients from a TB endemic region, who represent the specific population at increased risk for disease. Although both triglyceride and oxLDL levels were lower in the TB-DM group compared to DM, this might well be related to the duration and severity of DM disease as the majority of TB-DM patients were recently diagnosed diabetics compared to the DM alone group (S1 Table). Furthermore, TB was associated with wasting syndrome and therefore with low levels of many circulating metabolites in this patient population, including LDL [40]. As these patients were not merely at increased risk of TB at the moment of blood collection but had already developed active disease, it is not unlikely that oxLDL levels are decreased since onset of TB. Nonetheless, a clear dichotomy in oxLDL concentrations was visible based on triglyceride-status in TB-DM patients, implying that diabetes-associated dyslipidemia was a factor associated with increased oxLDL levels in this population.

Importantly, oxLDL-, but not acLDL-, induced foamy macrophage formation supported intracellular *Mtb* survival through lysosomal cholesterol accumulation and subsequent dysfunction. This effect was not limited to *Mtb* as similarly enhanced bacterial loads were observed for *Stm* and *M*. *bovis* BCG, which reside in different intracellular compartments compared to *Mtb* [57]. Pharmacological manipulation of intracellular cholesterol transport with U18666A confirmed that subcellular localization of cholesterol to lysosomes was essential to lysosomal dysfunction. Since foamy macrophages play an important role during progression of the TB granuloma [16, 18], our results suggest that increased levels of oxLDL could contribute to the enhanced TB susceptibility in DM patients.

Our findings are in line with earlier studies that reported increased levels of oxLDL in DM patients [8–11]. Both hyperglycemia and dyslipidemia contribute to the generation of free radicals and oxidative stress during chronic DM [58, 59], which can lead to the pathological modification of proteins and lipids involved in foam cell formation and atherosclerosis, such as oxLDL. Additionally, DM and hyperglycemia are associated with increased expression of oxLDL scavenger receptors CD36 [60–62], SR-A [62, 63] and LOX-1 [62, 64], and macrophages from type 2 diabetics showed higher uptake of oxLDL [65]. Similar to DM, TB has been demonstrated to result in increased oxidative stress and a systemic decrease in antioxidant capacity, *e*.*g*. reduced levels of glutathione [66–69]. *Mtb* infection increased CD36 expression *in vitro* [19] and CD36-mediated uptake of surfactant lipids has been reported to support *Mtb* growth [20]. In contrast, a recent paper did not find a role for CD36-mediated macrophage lipid droplet formation in *Mtb* control [70], which could indicate that not simply the presence of lipid droplets but rather the specific composition and/or localization of the intracellular lipids is most important for their effect on *Mtb* intracellular survival, similar to what we observed here when comparing acLDL and oxLDL.

At the functional level, oxLDL treatment displayed potential to inhibit macrophage antigen presentation to CD4^+^ T cells, which could in principal lead to impaired activation of adaptive immune responses. While their phagocytic capacity was largely unaffected, oxLDL-treatment macrophages showed an overall decreased cytokine production in response to *Mtb*. These results were somewhat surprising, as oxLDL has been associated with increased inflammation during atherosclerosis [71] and non-alcoholic steatohepatitis (NASH) [72–74], including activation of the NLRP3 inflammasome and subsequent secretion of IL-1β by macrophages [38, 75]. However, in these studies oxLDL treatment was often accompanied by secondary factors which may be required for the observed pro-inflammatory responses, such as macrophage apoptosis, circulating anti-oxLDL immune complexes or the formation of intralysosomal cholesterol crystals. In agreement with our own observations, several studies reported diminished inflammatory responses of oxLDL-treated macrophages after stimulation with TLR ligands [76–78]. These divergent results could be related to study-specific differences in experimental setup, including variations in species, cell types, stimulations and degree of LDL oxidation. Additionally, oxLDL was reported to induce a long-lasting pro-inflammatory phenotype in monocytes through epigenetic changes, which possibly did not occur in our experiments due to their relatively short timeframe or lack of restimulation [79, 80].

Hypercholesterolemia has been implicated in increasing the risk of developing TB [81–83], and cholesterol catabolism is needed for mycobacterial persistence and growth [84, 85]. For this reason, most studies on foamy macrophage induction by mycobacteria have focused on the relatively long-term nutritional benefits of intracellular lipid accumulation [20, 86]. The results presented in this manuscript demonstrate that pathologically modified lipids also directly interfere with macrophage antimicrobial capacities, providing a novel perspective on the importance of foam cell formation during TB. These findings are corroborated by a study which demonstrated that *M*. *smegmatis* and *M*. *bovis* BCG blocked phagolysosomal fusion by inducing an NPC-like phenotype in infected macrophages [39]. Additionally, macrophage cholesterol depletion restored halted phagosome maturation during *M*. *avium* infection [87]. Drugs which target host cholesterol metabolism can therefore have potential for TB host directed treatment, and *e*.*g*. statins have shown promise as adjunctive anti-mycobacterial therapy both *in vitro* and *in vivo* [88–92]. Furthermore, our results suggest that oxLDL treatment supports mycobacterial survival through interference with phagolysosomal trafficking and/or fusion. Lysosomal lipid accumulation has been reported to influence these processes in several ways. Late endosomal transport is mediated by the lysosomal protein ORP1L, which modulates the interaction between Rab GTPases and their effectors, motor protein complexes and the ER through conformational changes induced by fluctuations in intraluminal cholesterol levels [93, 94]. Furthermore, abnormal sphingolipid storage due to *NPC1*-deficiency or U18666A treatment was shown to disrupt lysosomal Ca^2+^ homeostasis, blocking vesicle transport and fusion [30, 46]. Finally, several studies have reported that lysosomal storage disorders interfere with the autophagic system [31, 32], which might be reflected by the increased LC3-II levels detected in oxLDL- and U18666A-treated macrophages during *Mtb* infection. Although pharmacological modulation of these pathways did not ameliorate the oxLDL-induced effect on *Mtb* control, their involvement should not yet be excluded as the phenotype induced by oxLDL was practically irreversible in our experimental setup.

Our study might have had a number of limitations. Firstly, the oxLDL used throughout this manuscript was generated by copper-induced oxidation of native LDL, which is sometimes referred to as extensively oxidized LDL in literature due to its high oxidation grade [6]. It is generally believed that naturally occurring oxLDL is composed of less extensively oxidized variants as abundantly oxidized LDL would be rapidly cleared from the circulation. Therefore, it is possible that the phenotypes observed in our experiments are more extreme than would have occurred using naturally oxidized LDL. However, the precise composition of physiological oxLDL is still uncertain as accurate characterization of isolated oxLDL is technically challenging. As LDL oxidation mostly occurs in the subendothelial space during atherosclerosis, locally generated oxidized species might be of greater importance for disease than circulating oxLDL. Regardless, it would be of interest to investigate the effects of minimally modified LDL (mmLDL), a variant which is believed to be more similar to naturally occurring oxLDL [6], on macrophage *Mtb* infection. Secondly, oxLDL was applied at a concentration of 25 μg/ml for the majority of the experiments, which is at the high end of what has been physiologically observed [95–97]. However, oxLDL treatment times were relatively short compared to what can be expected *in vivo*, and low levels of oxLDL (1 μg/ml) were already sufficient to increase mycobacterial load during this period. Thirdly, oxLDL is a complex particle consisting of hundreds of phospholipids, triglycerides and cholesteryl esters, which vary in terms of composition and susceptibility to oxidation and therefore have different intracellular effects [12]. It would be of great interest to study whether specific oxidized lipids or proteins are required for the observed oxLDL phenotype. Finally, although not within the scope of this study and technically challenging, it would be important to validate these findings in a disease model for translation to *in vivo* settings, e.g. using monocytes isolated from DM patients.

In conclusion, oxLDL treatment of human macrophages supports *Mtb* intracellular survival as a result of lysosomal dysfunction, providing a proof of concept for a contribution of increased levels of oxLDL as a potential risk factor for TB development during DM. While we previously demonstrated that hyperglycemia alone did not directly influence outcome of macrophage *Mtb* infection [98], we postulate that elevated lipid levels, which are associated with DM, can be in involved in TB-DM pathogenesis [40]. These findings pave the way for further research, including the use of LDL-lowering drugs such as statins or antioxidant drugs as part of the DM-treatment regimen for the reduction of the risk of TB.

## Materials and methods

### Ethics statement/Patient population and plasma oxLDL measurements

The patient population was previously used in an extensive lipid profiling analysis using H^+^-NMR spectroscopy as part of an EU-funded collaborative project, TANDEM [99], of which details regarding patient inclusion were reported earlier [40]. From this population plasma samples of 20 healthy endemic controls, 20 TB patients, 20 DM patients and 20 TB-DM patients were selected at random for oxLDL determination. Plasma oxLDL levels were measured by sandwich ELISA according to manufacturer’s instructions (Mercodia AB, Uppsala, Sweden). One TB-DM patient was excluded post-hoc due to the presence of clinical evidence suggestive of type 1 diabetes, while all other DM patients suffered from type 2 diabetes. This study was approved by the Health Research Ethics Committee of the University of Stellenbosch, and conducted according to the Helsinki Declaration and International Conference of Harmonization guidelines. Written informed consent was obtained from all participants.

### Reagents and antibodies

Primary antibodies against LAMP1, LAMP2, Cathepsin D, Cathepsin L, p62, galectin-3 and secondary goat anti-mouse IgG (Alexa Fluor 647) were purchased from Abcam (Cambridge, UK). LC3A/B was from Cell Signaling (Bioke, Leiden, The Netherlands), actin-HRP from Santa Cruz Biotechnology (Santa Cruz, CA, USA), CD86-Alexa700 and HLA-DR-PeCy5 from BD Biosciences (Erembodegem, Belgium) and CD80-BV650, CD14-FITC and CD163-Alexa647 were bought from Biolegend (ITK diagnostics, Uithoorn, The Netherlands). NDP52 (CALCOCO2), secondary goat anti-rabbit IgG (Alexa Fluor 647) and HRP-conjugated antibodies reactive with mouse and rabbit were purchased from Thermo Fisher Scientific (Merelbeke, Belgium).

### LDL isolation

LDL was isolated from the serum of healthy volunteers by density gradient ultracentrifugation [100]. Blood was collected in clot activator tubes and clotted for 90 minutes at room temperature. Serum was obtained after 10 minutes of centrifugation at 1,500 *g*. EDTA was added to a final concentration of 1 mM, after which serum density was adjusted to 1.21 g/l by addition of solid potassium bromide and gentle stirring. The resulting serum solution was distributed over 13.7 ml UltraClear ultracentrifuge tubes (Beckman Coulter, Woerden, The Netherlands) and a density gradient was prepared by overlaying it with potassium bromide solutions of decreasing concentrations (1.063 g/l, 1.019 g/l, 1.0063 g/l) in PBS supplemented with 0.3 mM EDTA (pH 7.4) using a wide bore pipette tip. The serum was then centrifuged at 40,000 RPM for 20 h at 4°C in a SW41 Ti swinging bucket rotor (Optima LE-80K, Beckman Coulter). After centrifugation the tubes were carefully removed from the rotor and the LDL fraction was aspirated using a glass Pasteur pipette. The LDL was dialyzed against PBS at 4°C for 16 h during which the buffer was refreshed three times. The protein concentration of LDL was determined using a BCA kit according to the manufacturer’s instructions (Pierce, Thermo Fisher Scientific).

### Generation of oxLDL and acetylated LDL (acLDL)

OxLDL was generated by copper oxidation of native LDL. Copper sulfate was added to 200 μg/ml LDL in PBS at a final concentration of 5 μM and incubated for 20 h at 37°C in the dark. The reaction was stopped by addition of 0.2 mM EDTA and oxLDL was then dialyzed against PBS containing 1 mM EDTA at 4°C for 24 h during which the buffer was refreshed three times. To produce acLDL, LDL was acetylated according to the protocol by Fraenkel-Conrat *et al*. [101]. An equal volume of saturated sodium acetate was added to 1 mg/ml of LDL and stirred at 4°C until cold. During the following hour acetic anhydride was added in 2 μl aliquots until 1.5x the mass of LDL was added in total. The mixture was stirred for another 30 minutes after the last aliquot was added. The acLDL was then dialyzed against PBS containing 1 mM EDTA at 4°C for 24 h during which the buffer was refreshed three times. Finally, the modified lipoproteins were concentrated to 1 mg/ml using 100 kDa Amicon Ultracel centrifugal filter units (Merck Millipore, Amsterdam, The Netherlands).

### Macrophage differentiation and foam cell generation

CD14^+^ monocytes were isolated from buffy coats of healthy blood bank donors by positive selection using an autoMACS Pro Separator (Miltenyi Biotec BV, Leiden, The Netherlands). Donors were not part of an already-existing collection. Monocytes were differentiated into macrophages by addition of 50 ng/ml macrophage-colony stimulating factor (M-CSF) (Miltenyi Biotec) during culture for 6 days at 37°C/5% CO_2_ [102]. Cells were cultured in RPMI-1640 medium with L-glutamine, without glucose and sodium bicarbonate (Sigma-Aldrich Chemie BV, Zwijndrecht, the Netherlands), supplemented with 5 mM D-glucose, 2 g/l sodium bicarbonate, 10% fetal bovine serum, 100 units/ml penicillin and 100 μg/ml streptomycin. After differentiation macrophages were harvested by trypsinization and seeded in multi-well plates. As a quality control, macrophages were stained for surface expression of CD14 and CD163 and acquired on a BD LSRFortessa flow cytometer (BD Biosciences) (S1A Fig). To generate foam cells, macrophages were treated with various concentrations of oxLDL overnight. PBS, native LDL and/or acLDL were used as controls. Foam cell formation was confirmed by Oil Red O staining. Macrophages were fixed for 30 minutes in 4% paraformaldehyde and subsequently stained with a filtered work solution of Oil Red O (Sigma-Aldrich) in isopropanol (0.3% Oil Red O in 60% isopropanol) for 20 minutes. Afterwards, the red stain was dissolved in 4% NP-40 in isopropanol and quantified by measuring the optical density (OD) at 520 nm using a iMark Microplate Absorbance Reader (Bio-Rad, Veenendaal, The Netherlands).

### *Mtb* H37Rv infection and cytokine measurements

*Mtb* H37Rv cultures were grown to mid-log phase in Middlebrook 7H9 liquid medium (Difco, BD Biosciences) supplemented with albumin/dextrose/catalase (ADC) (BBL, BD Biosciences). Bacterial concentrations were determined by measuring culture optical density at 600 nm. Macrophages were infected with H37Rv at a multiplicity of infection (MOI) of 10:1 for 1 h at 37°C, after which the cells were washed twice with medium containing 30 μg/ml gentamicin and cultured overnight in fresh medium containing 5 μg/ml gentamicin. Infected cells were lysed either directly after infection or at 4, 24, 48, 72 or 144 h post-infection using 0.05% Triton X-100 and a dilution series of the lysates was plated on 7H10 square agar plates (Difco, BD Biosciences) supplemented with oleate/albumin/dextrose/catalase (OADC) (BBL, BD Biosciences). Colony-forming units (CFU) were determined after 2–3 weeks of incubation at 37°C. From some experiments supernatants were harvested and filtered for determination of IL-1β, IL-6, TNF-α (Invitrogen, Thermo Fisher Scientific) and IL-10 (Sanquin, Amsterdam, The Netherlands) by ELISA or for testing using a Human Cytokine/Chemokine Immunology Multiplex Assay (Merck Millipore, Amsterdam, the Netherlands) according to their manufacturers’ instructions.

### Phagocytosis assay

To quantify phagocytic capacity, fluorescent polystyrene particles (Fluoresbrite YG carboxylate microspheres) (Polysciences, Hirschberg an der Bergstrasse, Germany) were used as described by Leclerc *et al* [103]. Macrophages were incubated with fluorescent beads in a ratio of 10 beads to 1 cell for 90 min at 37°C. Cells were subsequently harvested by gentle scraping and resuspended in a 1:1 mixture of culture medium and Trypan Blue, and internalization of the beads was quantified by acquisition on a BD Accuri C6 flow cytometer (BD Biosciences). Non-internalized bead fluorescence was quenched by Trypan Blue and detected in the FL-3 channel (red), whereas internalized beads were detected in the FL-1 channel (green). Analysis was performed using Flowjo software (version 10.1, Tree Star Inc, Ashland, OR).

### Antigen presentation assay

HLA-DR2/HLA-DR3-postive macrophages were harvested, seeded in 96-well plates at 2,500 cells/wells and treated with PBS, 25 μg/ml oxLDL or native LDL. The following day the cells were washed once in assay medium (IMDM with 10% human serum) and HLA class II restricted CD4^+^ T cell clones were added at a ratio of 4:1 together with a dilution series of their specific cognate peptide (R2F10 clone: HLA-DR2 restricted, reactive with *Mycobacterium leprae* (*Mlep*) hsp65; Rp15 1–1: HLA-DR3 restricted, reactive with *Mtb* and *Mlep* hsp65) or 1.25 μg/ml purified protein derivative (PPD) (Staten Serum Institute, Copenhagen, Denmmark) [104, 105]. Medium was used as negative control. Macrophages and T cells were co-cultured for 3 days at 37°C/5% CO_2_, and tritium-thymidine was added for the last 16 h of culture after which the cells were harvested and tritium-thymidine incorporation was measured using a Microbetaplate counter (Wallac, Turku, Finland). Furthermore, macrophages were stained for surface expression of CD86, CD80 and HLA-DR and analyzed on a BD LSRFortessa flow cytometer (BD Biosciences).

### Western blotting

For analysis of lysosomal and autophagy-related proteins, (*Mtb*-infected) macrophages were lysed for 5 minutes using a buffer containing 3% SDS, 4 mm glycerol, 100 mM Tris-HCl (pH 6.8) containing protease inhibitors (Roche, Woerden, The Netherlands) and the resulting lysates were boiled for 10 min at 95°C. Protein concentrations were determined by bicinchoninic acid assay (Pierce, Thermo Fisher Scientific) and equal amounts were mixed with 4x Laemmli buffer before loading on a 4–20% Mini-PROTEAN TGX precast protein gel (Bio-Rad). After separation, proteins were transferred onto a polyvinylidene fluoride membrane and blocked for 1 h in Tris-buffered saline/2.5% Tween-20 containing 5% non-fat dry milk and subsequently probed with primary antibodies overnight at 4°C. Membranes were incubated with horseradish peroxidase-conjugated secondary antibodies (reactive against mouse or rabbit) for 2 h at room temperature before visualization by Amersham Enhanced Chemiluminescence Western Blotting Detection kit (GE Healthcare, Hoevelaken, The Netherlands). Blots were quantified using Image J (NIH, Bethesda, MD, USA) and proteins were normalized versus actin.

### Confocal microscopy

For confocal microscopy, macrophages were seeded in black poly-d-lysine coated glass 96-well plates (MatTek Corporation, Ashland, MA, USA). To stain lysosomes, macrophages were incubated with 75 nM Lysotracker Red or Deep Red (Thermo Fisher Scientific) at 37°C/5%CO_2_ for 1 h before fixation. Cells were fixed for 1 h in 1% EM-grade formaldehyde, followed by quenching with PBS/1.5 mg/ml glycine for 10 min and blocking in 5% human serum for 45 min, all at room temperature. For immunostaining, cells were permeabilized for 10 minutes with 0.1% Triton X-100 before blocking and subsequently stained with primary and secondary antibodies for 30 minutes each in the dark at room temperature. Finally, cells were stained with phalloidin-Alexa488 (Thermo Fisher Scientific) and/or LipidTOX Green (Thermo Fisher Scientific) for 30 min according to the manufacturers’ instructions, and/or 50 μg/ml Filipin complex from *Streptomyces filipinensis* (Sigma-Aldrich) for 2 h at room temperature in the dark. Lysotracker and filipin pictures were taken using a SP8WLL confocal microscope (Leica, Amsterdam, The Netherlands). Galectin-3 and NDP52 colocalization was visualized using a Dragonfly spinning-disk confocal microscope (Andor Technologies, Belfast, UK) equipped with 405, 488, 561 and 640nm lasers and a Zyla 4.2 sCMOS camera.

### Colocalization analysis

Macrophages were infected for 4 h with a DsRed-expressing *Mtb* H37Rv strain at a MOI of 10:1 and stained with Lysotracker Deep Red or primary antibodies for galectin 3 and NDP52 as described above. Lysotracker channel background was subtracted by rolling ball algorithm (20 pixel radius). All images were analyzed using CellProlifer 3.0.0 [106]. First, pictures were corrected for non-homogenous illumination if necessary. DsRed-*Mtb* were segmented by manual global thresholding with intensity-based declumping, and stained objects were segmented by adaptive two-class Otsu thresholding with upper and lower bounds to correct for individual cell-specific differences in background signal with intensity-based declumping. Then, the percentage of staining object overlap with individual DsRed-*Mtb* was calculated for each image and the average colocalization was calculated for each treatment condition.

### Macrophage viability assay

To assess cell viability after treatment and infection with H37Rv *Mtb*, macrophages were stained with 2 μg/ml propidium iodide (PI) (Sigma-Aldrich) and 2 μg/ml Hoechst 33342 (Sigma-Aldrich) in RPMI without phenol red and FCS for 5 min in the dark. Cells were subsequently imaged on a AF6000 fluorescence microscope (Leica) and pictures were taken at a 20x magnification. Pictures were processed and analyzed in Image J. First, the background was subtracted by rolling ball algorithm (20 pixel radius). Then, Hoechst- or PI-positive nuclei were segmented by Otsu thresholding and counted, from which the percentages of viable macrophages were calculated. Staurosporin (5 μM) (Sigma-Aldrich) was used as a positive control for cell death.

### Statistical analysis

Statistical significance was assessed by Kruskal-Wallis test with post-hoc Dunn’s test, or Wilcoxon signed rank test using GraphPad software (version 7.02, Prism, La Jolla, CA, USA) with post-hoc false discovery rate (FDR) correction for multiple comparisons when necessary. Statistical analysis of patients characteristics was performed in SPSS 23 (IBM, Armonk, NY, USA) by one-way ANOVA (reported *p*-values are the outcome of the *F*-test), independent samples t-test or chi-squared test.

## Supporting information

S1 Fig Macrophage phenotype and Oil Red O staining. Monocyte-derived macrophages were differentiated using M-CSF (50 ng/ml) for 6 days. (A) Histograms of the cell surface expression of CD14 and CD163 as determined by flow cytometry. Stained (blue) and unstained (blue) samples are displayed. Data shown are from one representative donor. (B) Macrophages were treated overnight with PBS control, LDL, acLDL or oxLDL at 25 μg/ml and stained for neutral lipids with Oil Red O. Staining was dissolved and quantified by measuring OD at 520 nm. Data are displayed as ΔOD520 versus PBS control. Individual donors are depicted as dots with group medians. Statistical significance was determined by Kruskal-Wallis test with post-hoc Dunn’s test. ** = p < 0.01. (C) Macrophages were treated with 25 μg/ml oxLDL or PBS control overnight and infected with *Mtb* H37Rv for 24 h. OxLDL-induced increased *Mtb* loads were normalized to PBS control and plotted versus the infectious load (MOI) as determined by CFU assay (n = 34; each dot represents one individual donor). Kendall tau correlation and associated two-sided *p*-value are displayed.
(TIF)

S2 Fig oxLDL treatment diminished macrophage antigen presentation to a second CD4+ T cell clone and this was independent of cell surface expression of CD86, CD80 and HLA-DR. Primary human macrophages were treated with PBS control, native LDL or oxLDL (1, 10 or 25 μg/ml) overnight. (A) Macrophages were co-cultured for four days with the HLA-DR3-restricted CD4+ T cell Rp15 1–1 at a ratio of 1:4 and 0.1, 1 or 10 μg/ml of its cognate peptide or 1.25 μg/ml PPD. T cell proliferation was measured by tritium-thymidine incorporation during the last 24 h (n = 3). Data is represented as means with standard deviations. (B) Cell surface expression of CD86, CD80 and HLA-DR as determined by flow cytometry of macrophages treated overnight with PBS, native LDL or oxLDL (25 μg/ml). Stained (blue) and unstained (blue) samples are displayed. Data shown are from one representative donor (n = 3). (C) Primary human macrophages were treated overnight with PBS control (n = 15), acLDL (n = 9), oxLDL (n = 15) or native LDL (n = 6) (25 μg/ml) and subsequently infected with *Mtb* H37Rv at a MOI of 10:1. Cells were lysed directly after 1 h of infection and bacterial load was determined by CFU assay to determine *Mtb* uptake. Results were normalized versus PBS control and depicted as group medians with 95% confidence intervals.
(TIF)

S3 Fig U18666A and oxLDL increased protein markers of lysosomes and autophagy in *Mtb*-infected macrophages without affecting cell viability. Primary human macrophages were treated with PBS control, acLDL or oxLDL (25 μg/ml) for 24 h prior to infection with *Mtb* H37Rv at a MOI of 10:1. (A) Representative Western Blot result of Cathepsin D protein levels from macrophages treated with bafilomycin A1 (10 nM) or DMSO, showing protein bands of both the mature heavy chain (34 kDa) and the processing intermediate pro-cathepsin D (48 kDa) after 30 and 900 seconds of exposure time. Pro-cathepsin D levels were first normalized to actin and subsequently versus PBS control (n = 4). (B) Western blot analysis of lysosomal and autophagy markers in macrophages co-treated with PBS, oxLDL or acLDL (25 μg/ml) and U18666A (3 μg/ml), bafilomycin A1 (10 nM) or DMSO control during 24 h of H37Rv *Mtb* infection. Data shown is from one representative donor (n = 2). (C) *Mtb*-infected macrophages were stained with Hoechst and PI to determine cell viability. Staurosporin (5 μM) and PBS were used as positive and negative control for cell death. (D) Percentages of viable cells (Hoechts+/PI-). Data are displayed as means with standard deviations (n = 4).
(TIF)

S4 Fig Kinetic analysis of intracellular *Mtb* survival and associated cytokine responses in human macrophages. Primary human macrophages were treated with PBS control, acLDL or oxLDL (25 μg/ml) overnight and subsequently infected with *Mtb* H37Rv at a MOI of 10:1. Cells were lysed at 0 (uptake), 4, 24, 48, 72 and 144 h post-infection for CFU analysis (n = 7). (A) Intracellular *Mtb* loads are depicted as fraction of uptake in Tukey’s boxplots for each time point and condition: PBS (white), acLDL (grey) and oxLDL (red). (B) Supernatants were harvested at each time point post-infection and cytokine concentrations were determined by multiplex assay. Levels of IL-10, IL-6, TNF-α, IL-8, IL-1RA, CXCL10, IFNα2, CCL2, CCL3, CCL4, G-CSF, VEGF and GM-CSF (pg/ml) are depicted in Tukey’s boxplots for each time point and condition: PBS (green), acLDL (purple) and oxLDL (red). Group medians are shown as dashed lines. Statistical significance was determined by Wilcoxon signed rank test with post-hoc FDR correction. *p* < 0.05 for * = PBS vs oxLDL, # = oxLDL vs acLDL, ‡ = PBS vs acLDL.
(TIF)

S1 Table Patient clinical characteristics according to disease group (n = 79). Data is presented as percentage of total (%) or mean ± SD, *Point-of-care measurements, †lab measurements, ^1^data available from 12/19 patients, ^2^data available from 12/20 patients, ^3^data available from 16/19 patients.
(DOCX)
