# Supplementary figures and images for: Oxidized low-density lipoprotein (oxLDL) supports Mycobacterium tuberculosis survival in macrophages by inducing lysosomal dysfunction
Source: PLoS Pathog. 2019 Apr 18;15(4):e1007724. doi: 10.1371/journal.ppat.1007724 (PMC6490946; doi:10.1371/journal.ppat.1007724)

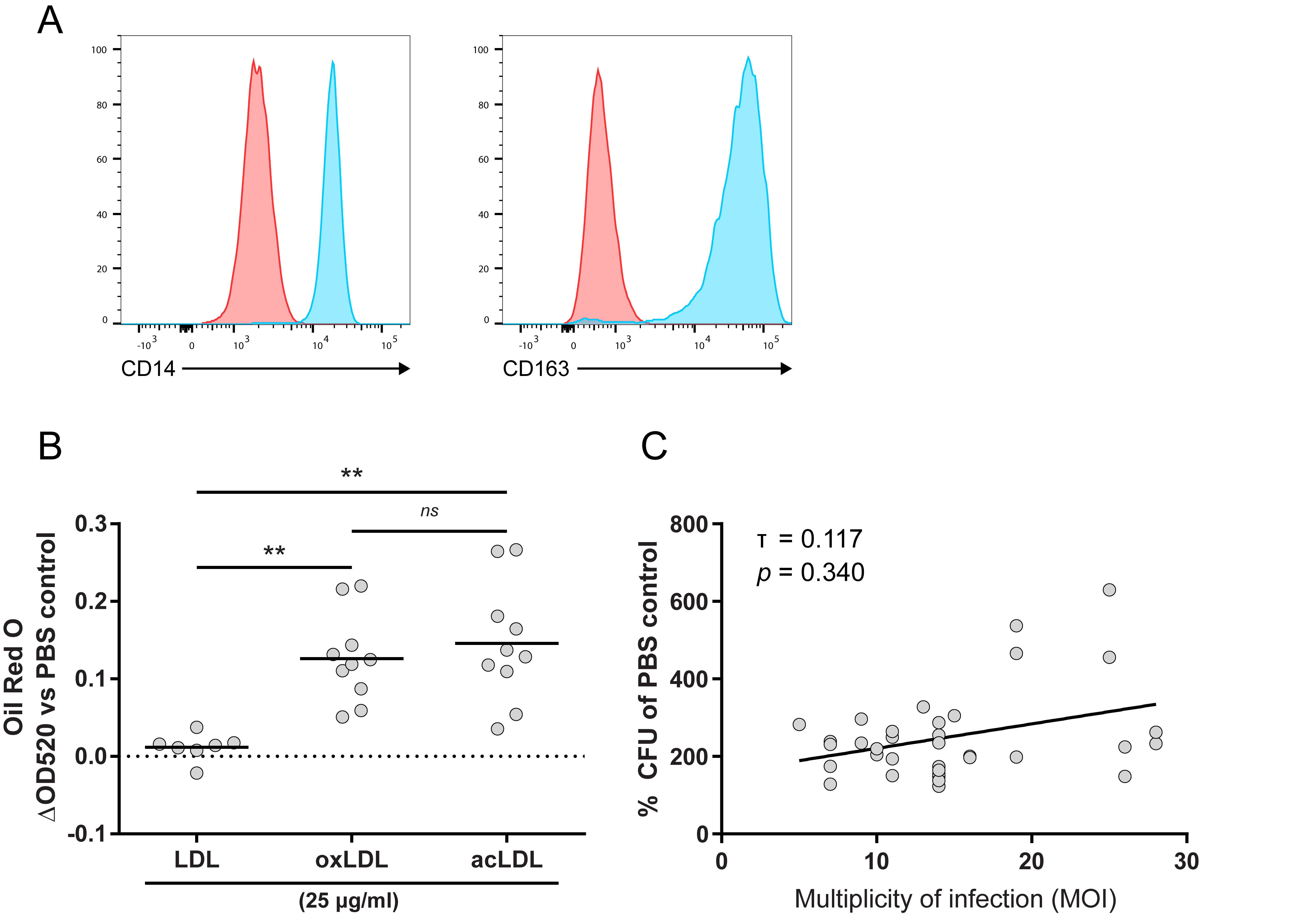

Supplement: S1 Fig — Monocyte-derived macrophages were differentiated using M-CSF (50 ng/ml) for 6 days. (A) Histograms of the cell surface expression of CD14 and CD163 as determined by flow cytometry. Stained (blue) and unstained (blue) samples are displayed. Data shown are from one representative donor. (B) Macrophages were treated overnight with PBS control, LDL, acLDL or oxLDL at 25 μg/ml and stained for neutral lipids with Oil Red O. Staining was dissolved and quantified by measuring OD at 520 nm. Data are displayed as ΔOD520 versus PBS control. Individual donors are depicted as dots with group medians. Statistical significance was determined by Kruskal-Wallis test with post-hoc Dunn’s test. ** = p < 0.01. (C) Macrophages were treated with 25 μg/ml oxLDL or PBS control overnight and infected with Mtb H37Rv for 24 h. OxLDL-induced increased Mtb loads were normalized to PBS control and plotted versus the infectious load (MOI) as determined by CFU assay (n = 34; each dot represents one individual donor). Kendall tau correlation and associated two-sided p-value are displayed. (TIF) [file ppat.1007724.s001.tif]

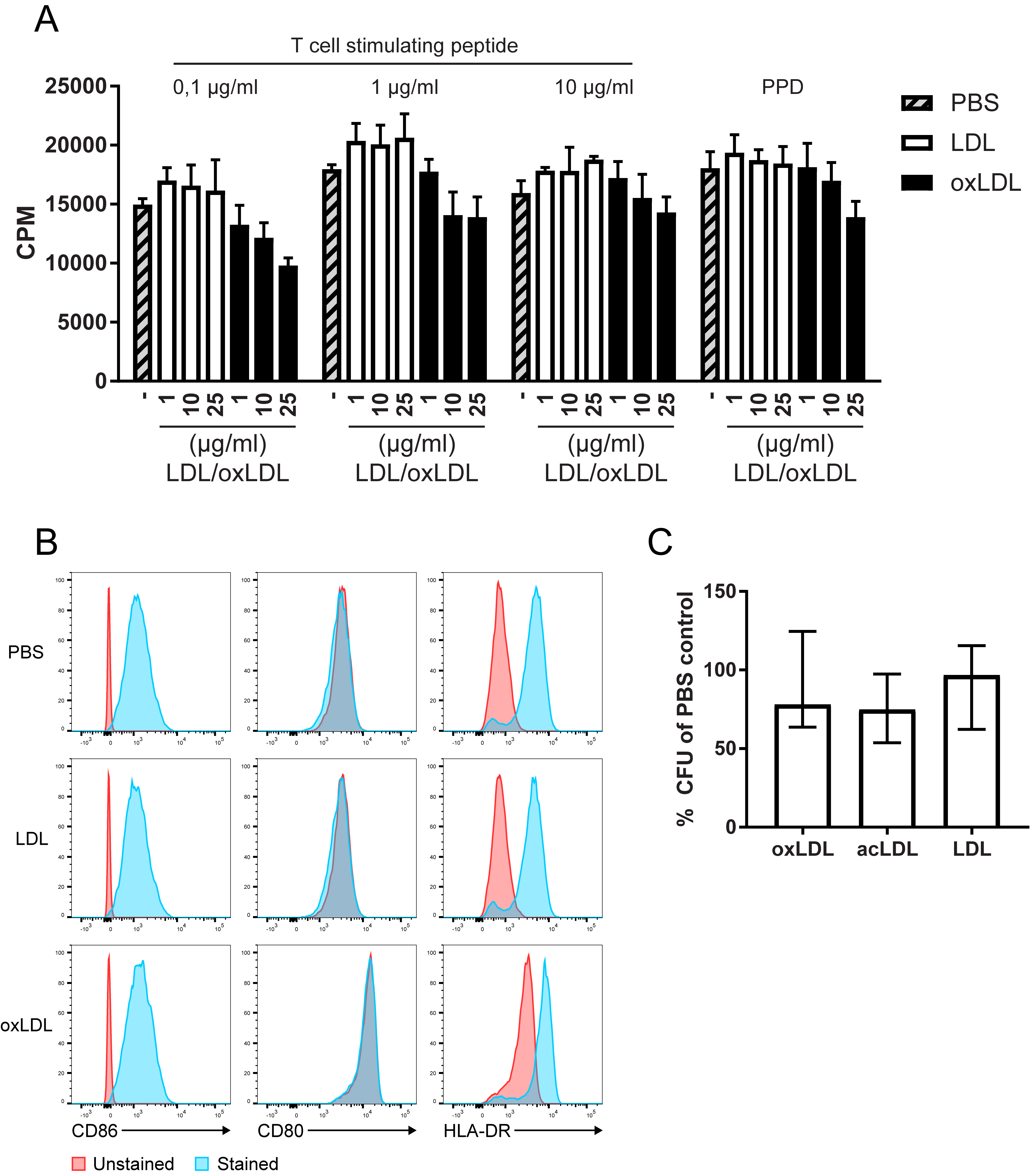

Supplement: S2 Fig — Primary human macrophages were treated with PBS control, native LDL or oxLDL (1, 10 or 25 μg/ml) overnight. (A) Macrophages were co-cultured for four days with the HLA-DR3-restricted CD4+ T cell Rp15 1–1 at a ratio of 1:4 and 0.1, 1 or 10 μg/ml of its cognate peptide or 1.25 μg/ml PPD. T cell proliferation was measured by tritium-thymidine incorporation during the last 24 h (n = 3). Data is represented as means with standard deviations. (B) Cell surface expression of CD86, CD80 and HLA-DR as determined by flow cytometry of macrophages treated overnight with PBS, native LDL or oxLDL (25 μg/ml). Stained (blue) and unstained (blue) samples are displayed. Data shown are from one representative donor (n = 3). (C) Primary human macrophages were treated overnight with PBS control (n = 15), acLDL (n = 9), oxLDL (n = 15) or native LDL (n = 6) (25 μg/ml) and subsequently infected with Mtb H37Rv at a MOI of 10:1. Cells were lysed directly after 1 h of infection and bacterial load was determined by CFU assay to determine Mtb uptake. Results were normalized versus PBS control and depicted as group medians with 95% confidence intervals. (TIF) [file ppat.1007724.s002.tif]

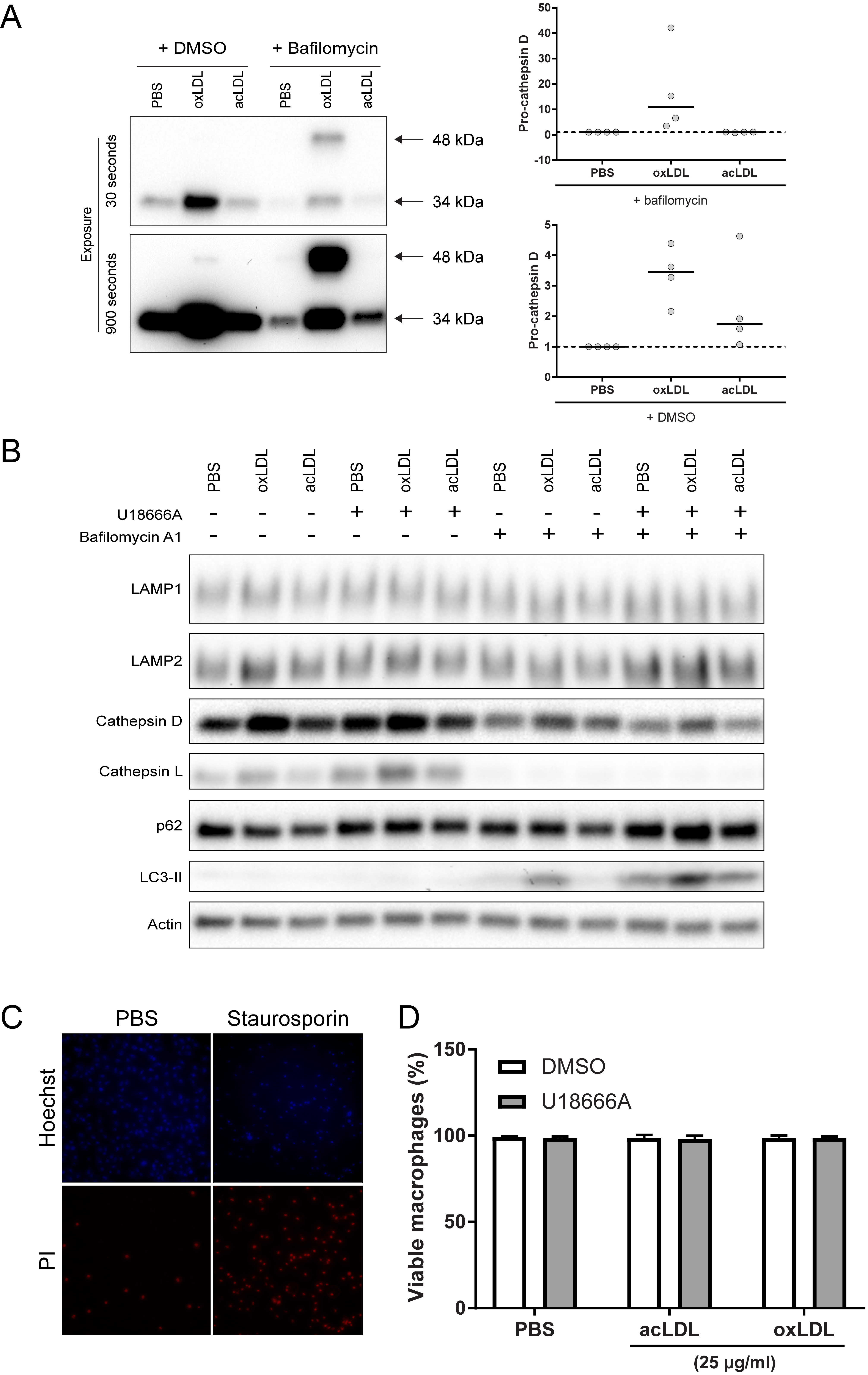

Supplement: S3 Fig — Primary human macrophages were treated with PBS control, acLDL or oxLDL (25 μg/ml) for 24 h prior to infection with Mtb H37Rv at a MOI of 10:1. (A) Representative Western Blot result of Cathepsin D protein levels from macrophages treated with bafilomycin A1 (10 nM) or DMSO, showing protein bands of both the mature heavy chain (34 kDa) and the processing intermediate pro-cathepsin D (48 kDa) after 30 and 900 seconds of exposure time. Pro-cathepsin D levels were first normalized to actin and subsequently versus PBS control (n = 4). (B) Western blot analysis of lysosomal and autophagy markers in macrophages co-treated with PBS, oxLDL or acLDL (25 μg/ml) and U18666A (3 μg/ml), bafilomycin A1 (10 nM) or DMSO control during 24 h of H37Rv Mtb infection. Data shown is from one representative donor (n = 2). (C) Mtb-infected macrophages were stained with Hoechst and PI to determine cell viability. Staurosporin (5 μM) and PBS were used as positive and negative control for cell death. (D) Percentages of viable cells (Hoechts+/PI-). Data are displayed as means with standard deviations (n = 4). (TIF) [file ppat.1007724.s003.tif]

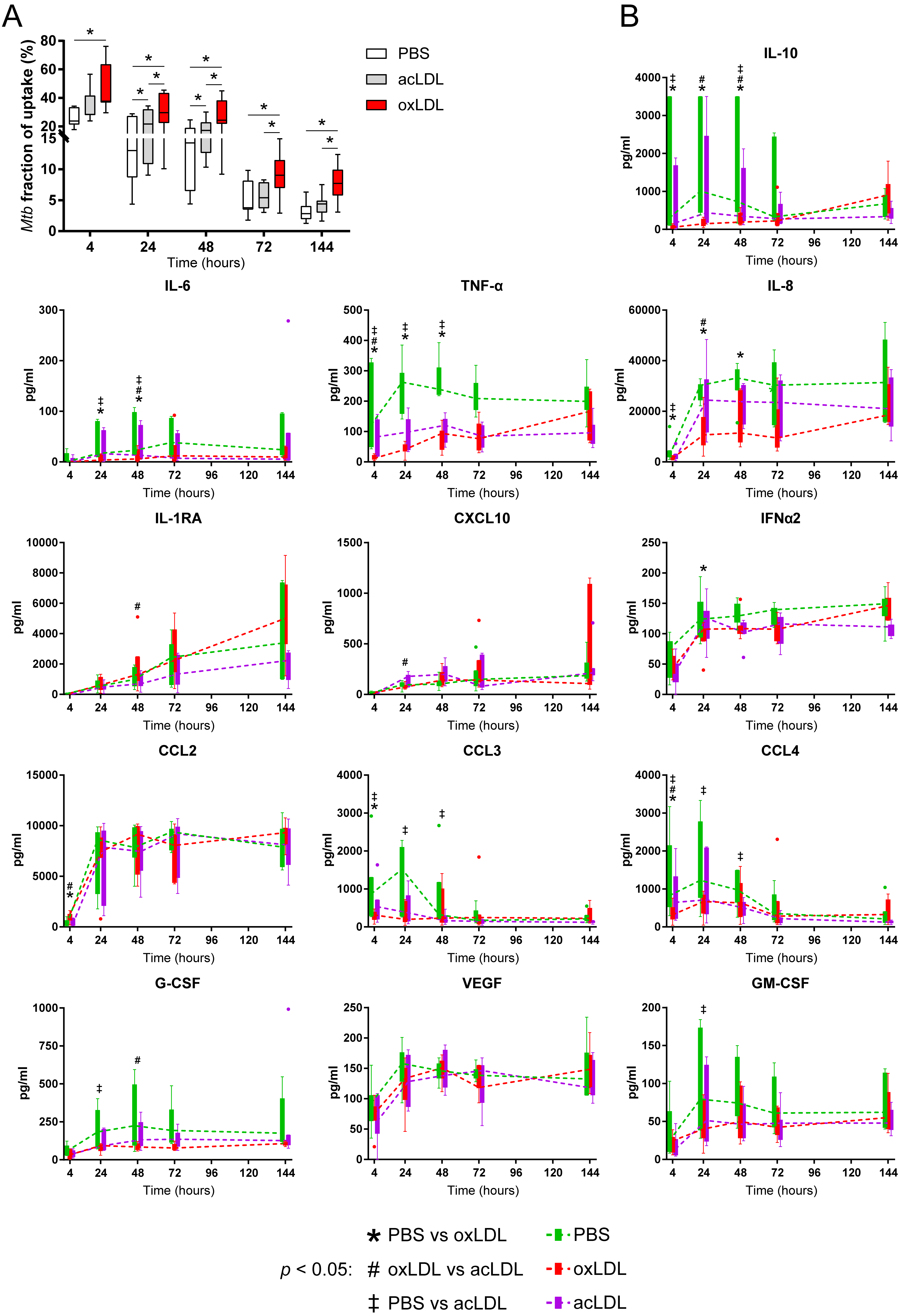

Supplement: S4 Fig — Primary human macrophages were treated with PBS control, acLDL or oxLDL (25 μg/ml) overnight and subsequently infected with Mtb H37Rv at a MOI of 10:1. Cells were lysed at 0 (uptake), 4, 24, 48, 72 and 144 h post-infection for CFU analysis (n = 7). (A) Intracellular Mtb loads are depicted as fraction of uptake in Tukey’s boxplots for each time point and condition: PBS (white), acLDL (grey) and oxLDL (red). (B) Supernatants were harvested at each time point post-infection and cytokine concentrations were determined by multiplex assay. Levels of IL-10, IL-6, TNF-α, IL-8, IL-1RA, CXCL10, IFNα2, CCL2, CCL3, CCL4, G-CSF, VEGF and GM-CSF (pg/ml) are depicted in Tukey’s boxplots for each time point and condition: PBS (green), acLDL (purple) and oxLDL (red). Group medians are shown as dashed lines. Statistical significance was determined by Wilcoxon signed rank test with post-hoc FDR correction. p < 0.05 for * = PBS vs oxLDL, # = oxLDL vs acLDL, ‡ = PBS vs acLDL. (TIF) [file ppat.1007724.s004.tif]
