# Supplementary material for: Oxidized low-density lipoprotein (oxLDL) supports Mycobacterium tuberculosis survival in macrophages by inducing lysosomal dysfunction
Source: PLoS Pathog. 2019 Apr 18;15(4):e1007724. doi: 10.1371/journal.ppat.1007724 (PMC6490946; doi:10.1371/journal.ppat.1007724)
Supplement: S1 Table — Data is presented as percentage of total (%) or mean ± SD, *Point-of-care measurements, †lab measurements, 1data available from 12/19 patients, 2data available from 12/20 patients, 3data available from 16/19 patients. (DOCX) [file ppat.1007724.s005.docx]

| **Table S1.**  Patient clinical characteristics according to disease group (n = 79) | | | | | |
| --- | --- | --- | --- | --- | --- |
|  | HC  n=20 | DM  n=20 | TB  n=20 | TB-DM  n=19 | *p*-value |
| Ethnicity: Coloured | 20/20 (100%) | 20/20 (100%) | 19/20 (95%) | 19/19 (100%) | 0.394 |
| Sex (male/female) | 10/10 | 7/13 | 11/9 | 11/8 | 0.482 |
| Age (years) | 37.3 ± 9.7 | 49.8 ± 12.4 | 45.7 ± 8.5 | 44.8 ± 10.3 | 0.003 |
| BMI (kg/m^2^) | 23.6 ± 7.0 | 31.2 ± 6.7 | 19.4 ± 3.0 | 20.7 ± 4.4 | < 0.001 |
| HbA1c (%) | 5.2 ± 0.3* | 10.0 ± 2.3* | 5.6 ± 0.3† | 8.8 ± 2.6† | < 0.001 |
| Random blood glucose (mmol/l) | 4.8 ± 1.0 | 14.3 ± 5.2 | 5.8 ± 1.3 | 8.6 ± 4.9^1^ | < 0.001 |
| Previous TB (>1 year ago) | na | 3/20 (15%) | 11/20 (55%) | 5/19 (26.3%) | 0.021 |
| Smoking (currently) | na | 5/20 (25%) | 17/20 (85%) | 14/19 (73.7%) | < 0.001 |
| Quantiferon positive | 13/19 (68.4%) | 15/19 (78.9%) | na | na | 0.461 |
| Time to positivity (days) | na | na | 8.3 ± 5.6^2^ | 7.8 ± 4.8^3^ | 0.794 |
| DM medication | na | 20/20 (100%) | na | 7/19 (36.8%) | 0.001 |
| Insulin |  | 12/20 (60%) |  | 2/19 (10.5%) | 0.003 |
| Metformin |  | 16/20 (80%) |  | 6/19 (31.6%) | 0.002 |
| Statins |  | 6/20 (30%) |  | 1/19 (5.3%) | 0.044 |
| Other |  | 4/20 (20%) |  | 2/19 (10.5%) | 0.412 |
| Years since DM diagnosis | na |  | na |  |  |
| <1 |  | 0/20 (0%) |  | 14/19 (73.7%) | < 0.001 |
| 1-5 |  | 6/20 (30%) |  | 1/19 (5.3%) | 0.044 |
| 6-15 |  | 8/20 (40%) |  | 3/19 (20.8%) | 0.093 |
| >15 |  | 6/20 (30%) |  | 1/19 (5.3%) | 0.044 |

Data is presented as percentage of total (%) or mean ± SD, *Point-of-care measurements, †lab measurements, ^1^data available from 12/19 patients, ^2^data available from 12/20 patients, ^3^data available from 16/19 patients
